# Visual inspection with acetic acid as a cervical cancer test: accuracy validated using latent class analysis

**DOI:** 10.1186/1471-2288-7-36

**Published:** 2007-07-31

**Authors:** Lynne Gaffikin, John A McGrath, Marc Arbyn, Paul D Blumenthal

**Affiliations:** 1 Cervical Cancer Prevention Program, JHPIEGO, Baltimore, USA; 2 Evaluation and Research Technologies for Health Incorporated, Stanford USA; 3 Department of Psychiatry, Johns Hopkins Medical Institution, Baltimore, USA; 4 Unit of Cancer Epidemiology, Scientific Institute of Public Health, Brussels Belgium; 5 Department of Obstetrics and Gynecology, Stanford University, Stanford, USA

## Abstract

**Background:**

The purpose of this study was to validate the accuracy of an alternative cervical cancer test – visual inspection with acetic acid (VIA) – by addressing possible imperfections in the gold standard through latent class analysis (LCA). The data were originally collected at peri-urban health clinics in Zimbabwe.

**Methods:**

Conventional accuracy (sensitivity/specificity) estimates for VIA and two other screening tests using colposcopy/biopsy as the reference standard were compared to LCA estimates based on results from all four tests. For conventional analysis, negative colposcopy was accepted as a negative outcome when biopsy was not available as the reference standard. With LCA, local dependencies between tests were handled through adding direct effect parameters or additional latent classes to the model.

**Results:**

Two models yielded good fit to the data, a 2-class model with two adjustments and a 3-class model with one adjustment. The definition of latent disease associated with the latter was more stringent, backed by three of the four tests. Under that model, sensitivity for VIA (abnormal+) was 0.74 compared to 0.78 with conventional analyses. Specificity was 0.639 versus 0.568, respectively. By contrast, the LCA-derived sensitivity for colposcopy/biopsy was 0.63.

**Conclusion:**

VIA sensitivity and specificity with the 3-class LCA model were within the range of published data and relatively consistent with conventional analyses, thus validating the original assessment of test accuracy. LCA probably yielded more likely estimates of the true accuracy than did conventional analysis with in-country colposcopy/biopsy as the reference standard. Colpscopy with biopsy can be problematic as a study reference standard and LCA offers the possibility of obtaining estimates adjusted for referent imperfections.

## Background

Cervical cancer, the second most commonly diagnosed cancer among women worldwide, can be a preventable disease. Although the Pap smear remains the most common screening test for cervical cancer, many less developed countries do not have adequate resources to implement cytology-based prevention programs. An alternative, low-cost test, visual inspection using acetic acid (VIA), has emerged for use in low-resource settings where it can be performed by auxiliary health professionals [1-3]. VIA is similar to colposcopy in that acetic acid is applied and any acetowhite lesion is visualized, although with VIA there is no magnification.

VIA accuracy studies have yielded a range of sensitivity and specificity values spanning from approximately 60 percent to over 90 percent [4-14]. While this range is narrower than observed for other tests including cytology (23% to 99% for sensitivity and 7% to 97% for specificity), it is important to investigate possible reasons for inter-study variability [15]. Some have questioned whether the variability of results across studies is due, at least in part, to imperfections with the reference standard used. For cervical cancer, the "gold" standard for establishing a diagnosis is biopsy [16]. The VIA studies cited above have involved a variety of reference standard measures. These include: 100 percent biopsy sampling, a combined colposcopy/biopsy reference standard for all participants, biopsy for colposcopically-suspicious lesions only, and colposcopy with histology only for women test-positive on all screening tests [i.e., visual inspection, Pap, human papilloma virus (HPV) and cervicography] [4-6,8,10,12-14]. Even among studies with similar reference standard measures, another source of variability across studies could be differences in the quality of the reference standard. Subjective (human) error may have affected the quality of colposcopy or the quality of tissue collection, slide fixing and biopsy interpretation which could have led to misclassification of the reference standard [17,18].

Most published studies on VIA involve use of conventional methods and a 2 × 2 table for assessing test accuracy (i.e., sensitivity and specificity). In recent years, several statistical methods have been used to evaluate new tests when no or an imperfect gold/reference standard is available [19-21]. LCA is a statistical technique, originally developed in the early 1950s, that allows for the accuracy of a new test to be assessed in the absence of a gold standard. It does this by using the statistical associations among various tests performed on the same individual to define unobserved (latent) disease. The likelihood of the relationship between latent disease, the new test under investigation and the other tests is then maximized to yield sensitivity and specificity estimates [22]. Historically, LCA has been used in biomedical applications to identify disease based on observable traits [23-25]. More recently, there has been increased interest in using LCA to evaluate diagnostic or screening tests [26-30].

The objectives of this analysis were two-fold: 1) to assess test accuracy using LCA assuming no gold standard and to compare those values with conventional estimates to explore the effect of any gold standard imperfections in calculating the latter; 2) to assess whether the assumption of independence between VIA and coloposcopy as a component of the gold standard reference test were met (a prerequisite for valid test accuracy assessment). The second objective corresponds to issues that have been raised regarding the appropriateness of using colposcopy as a reference standard for VIA accuracy studies as the two tests are similar in nature, both involving visual observation of the cervix after acetic acid wash [31].

## Methods

The dataset used in this exercise is a subset of data from a previously published, cross-sectional study [5]. All subjects participating in that study gave informed consent and the study was approved by the Institutional Review Board at Johns Hopkins Bayview Medical Center, Baltimore, USA.

The original study involved 2203 women, aged 25 to 55 years, who attended 15 primary-care clinics in two peri-urban areas near Harare, Zimbabwe between October 1996 and August 1997. Details of the original study design, data collection and biological sample collection procedures are available elsewhere [5]. All women enrolled in the study were offered and scheduled to receive four tests: VIA, a Pap smear, colposcopy with biospy (the latter when clinically indicated only) and a HPV DNA test [Hybrid Capture II (HC2), (Digene Corporation, Gaithersburg, USA].

Specially-trained nurse-midwives performed VIA using a 4 percent acetic acid dilution. A cytological specimen from the same woman was independently assessed by a local cytopathologist (and later reviewed by a board-certified, cytopathologist at the Johns Hopkins Bayview Medical Center, Baltimore, USA). In addition, HPV testing was independently performed for all women (at Johns Hopkins University, Bloomberg School of Public Health, Baltimore, USA) using the B probe of HC2 targeting 13 high-risk HPV types [32]. A colposcopic examination was performed by one of two local faculty gynecologists, blinded to all other test results, shortly after VIA testing and the cytology/HPV samples had been obtained. Almost all (97 percent) women received colposcopy. Biopsy was performed only for cases suspicious on colposcopy (n = 595).

In this study, analysis were performed on the subset of women (n = 2073) for whom all *four *test results (VIA, Pap, HPV and colposcopy/biopsy, here considered as one combined test) were available. A combined reference standard was developed incorporating biopsy results when available; negative colposcopy was accepted as a negative outcome when no biopsy was taken. Such a combined reference standard has been considered appropriate for cervical cancer test accuracy studies given ethical and other issues involved in performing biopsy for test-negatives [13]. The availability of colposcopy and/or biopsy results to form a combined reference standard for 97 percent of all study participants meant that *conventional *estimates of sensitivity/specificity could be calculated with an ignorable risk for verification bias. All tests were categorized into two levels for sensitivity/specificity estimates. Cervical intraepithelial neoplasia grade 2 or worse (CIN2+) on biopsy or high grade squamous intraepithelial lesion or worse (HGSIL+) on colposcopy was used as the cutoff point defining disease in all analyses. This threshold of disease was chosen since this is the severity level usually treated in Zimbabwe.

In the present analysis, LCA (as implemented in Latent Gold Version 3.0 software) was applied to four "manifest" variables (VIA, Pap smear, HC2, and colposcopy/biopsy) to construct a "latent" variable that could serve as a measure of the reference standard defining true disease [33]. Since LCA estimates the conditional probability of a given latent class (e.g., presence or absence of high grade lesions or worse) for each level of the observed variables, sensitivity and specificity in relation to the latent disease variable could be calculated. Using LCA, maximum-likelihood-based estimates of the standard errors of the various probability estimates were also calculated to derive confidence intervals. We then compared LCA-estimated sensitivity and specificity estimates to those conventionally derived from 2 × 2 tables with binomial standard errors (for VIA, Pap, and HC2 against colposcopy/biopsy as the reference standard).

A fundamental assumption of LCA is that the manifest variables are *locally independent*. That is, within (or local to) a given latent class, the manifest variables should be statistically unrelated to or independent from each other. Bivariate residuals, which reflect any remaining association among each pairing of manifest variables after estimation of the latent classes, indicate whether this assumption was in fact met.

Vermunt and Magidson suggest that residuals above 1.0 indicate possible local dependencies which can be adjusted for in LCA models through the introduction of "direct effects", representing the excess variation between two variables. Adding a direct effect increases the log-likelihood (LL) of the model, indicating better fit of the data to the model. The bivariate residual value approximates the increase in LL observed with the addition of the direct effect variable [33]. The software we used supports adjustment for local dependencies through the introduction of direct effects [34-36].

Model fit is also indexed by the likelihood ratio chi-square (L^2^) which *decreases *as the fit of the model improves. A significant p-value associated with the L^2 ^indicates that the manifest variables (here, the different tests) have associations with each other that are not accounted for by the model. LCA modeling continues with the addition of latent classes and/or adjustment for local dependencies until adequate fit is reached, as indicated by a non-significant p-value.

For our LCA, the tests were first categorized into three levels (Table 1) to increase the amount of information available to calculate the latent classes. Then, the tests were categorized dichotomously (as with the conventional test accuracy formula) to calculate sensitivity/specificity values as follows: VIA negative (normal, atypical/inflammation) versus VIA positive (abnormal, cancer); Pap negative [normal, inflammation, atypical squamous cells of uncertain significance (ASCUS), atypical glandular cells of uncertain significance (AGUS)] versus Pap positive [low grade squamous intraepithelial lesions (LGSIL), HGSIL, cancer]; HPV negative [< 1.0 relative light units (RLU) compared to control] versus HPV positive (> = 1 RLU compared to control); colposcopy/biopsy negative (normal, inflammation, pure HPV, LGSIL) versus colposcopy/biopsy positive (HGSIL, cancer). In this analysis, however, the latent class-derived reference standard was used as the measure of "true" disease. A preliminary 1-class model provided a baseline measure of the amount of association among the tests, as indicated by the log-likelihood (**LL**). Other LCA models were then generated to explain these associations and successively evaluated for their fit to the data.

With latent class modeling, it is often possible to produce several different models that adequately fit the data. In the present exercise, two such models were generated: one with two latent classes and one with three. There is no single statistical tool that can definitively support the selection of one model over the other and, therefore, final model selection must be based on knowledge of the biological processes under study, as well as the particular characteristics of the study sample. However, information criterion statistics such as the Akaike Information Criterion (**AIC**), available in Latent Gold Version 3.0, have been developed to provide an idea of the relative distance of two or more models from a theoretical best model [37]. The AIC is a function of the LL and the number of parameters (K) in the model: AIC = -2(LL) + 2K [38]. Given two or more fitted models on the same dataset, the one with the lower AIC value is considered better. From the equation for AIC, it is apparent that as LL increases, AIC decreases (gets better). Additionally, as the number of parameters (K) in a model increases, the AIC increases (gets worse). Thus, the AIC favors a more parsimonious model (lower K), all other things being equal.

**Table 1:** Trichotomous coding scheme for the four tests

Test	Low	Medium	High
VIA	Normal	Atypical	Abnormal, Cancer
Pap	Normal, Inflammation, Ascus, Agus	LGSIL	HGSIL, Cancer
HPV	< 1.0 RLU compared to control	>= 1.0 RLU and < 20.0 RLU compared to control	>= 20.0 RLU compared to control
Colposcopy/Biopsy*	Normal, Inflammation, Pure HPV	LGSIL	HGSIL, Cancer

* For subjects with both biopsy and colposcopy results, biopsy result was used.

## Results

Table 2 gives overall statistics for the final two best-fit LCA models (2-class and 3-class). As noted, while the simple 2-class model accounted for a large proportion of the variance, there still remained a significant amount of variance to be explained, as evidenced by the significant p value (4.5*10^-9^). Examination of the model bivariate residuals (= 10.03) revealed a local dependency between VIA and colposcopy/biopsy, indicating that significant association remained between these two variables after construction of the two latent classes (Table 3 provides the bivariate residuals for all models tested). A 2-class model with one adjustment for the residual association between VIA and colposcopy/biopsy was subsequently developed and results for this also appear in Table 2.

**Table 2:** Comparative statistics for various LCA models involving all four tests

Model	Parameters	LL	L^2^	p	AIC
1-class	8	-6592.39	1121.16	5.70E-188	13200.77
2-class	17	-6106.87	150.13	4.50E-09	12247.74
2-class with one adjustment	21	-6074.14	84.67	0.016	12190.28
2-class with two adjustments	25	-6065.57	67.54	0.12	12181.15
3-class with one adjustment	30	-6059.23	54.86	0.3	12178.47

**Table 3:** Bivariate residuals for various LCA models

	1-class (baseline)	2-class	2-class with adjustment for VIA * Colpo/Biop^1^	2-class with additional adjustment for Pap * Colpo/Biop	3-class with adjustment for VIA * Colpo/Biop
VIA * Pap	25.734	0.778	0.419	0.661	0.823
VIA * Colpo/Biop	46.100	10.027	0.000	0.000	0.000
VIA * HPV	42.092	0.549	0.775	0.299	0.267
Pap * Colpo/Biop	61.058	1.848	2.457	0.000	0.597
Pap * HPV	126.607	0.772	0.507	0.455	0.198
Colp/Biop * HPV	63.347	1.136	1.111	1.163	0.446

^1 ^Colpo/Biop = colposcopy/biopsy

**Figure 1:**
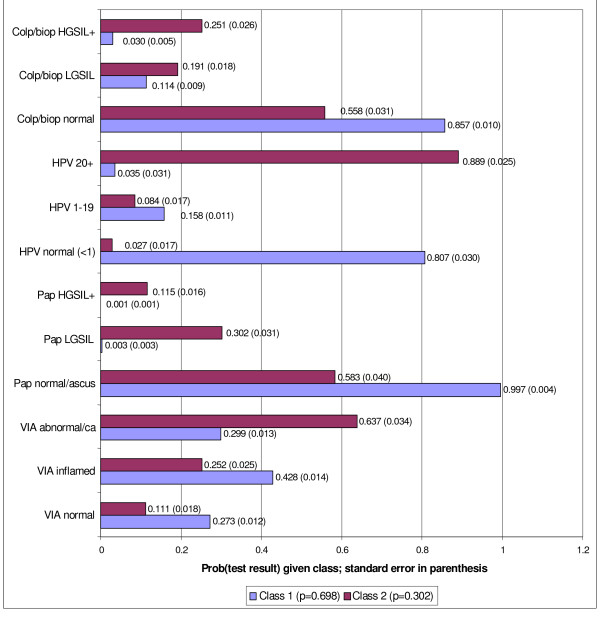
Probability of the Two Class LCA Model Test Result Conditional on the Latent Class*. *model probabilities generated from trichotomous results for all four tests.

Although this adjustment accounted for more variance (as seen by the increase in LL and decrease in L^2^), the overall model p value was still statistically significant from the 1-class, saturated model (p = 0.016), indicating more variance still needed to be explained. The 2-class model with one adjustment had a residual score of 2.4568 for the association of Pap smears with colposcopy/biopsy, meaning that these two tests were *also *locally dependent. Adding a second adjustment for this local dependency resulted in a non-significant p-value (0.12), indicating a good fit of the 2-class, two adjustment model to the data. Figure 1 shows the conditional probabilities of different test results for the 2-class model constructed from all four tests (with trichotomous categories) with two local dependency adjustments.

**Figure 2:**
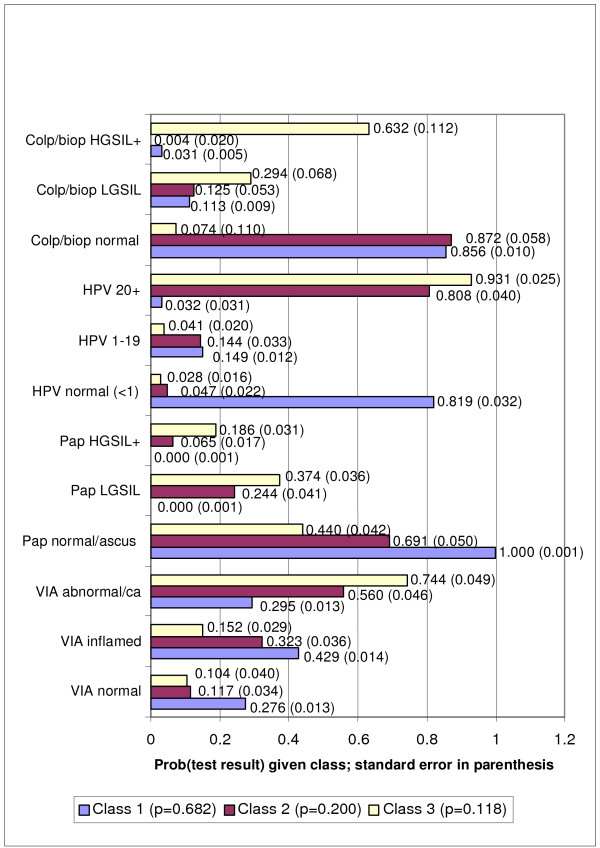
Probability of the Three Class LCA Model Test Result Conditional on the Latent Class*. *model probabilities generated from trichotomous results for all four tests.

The last row of Table 2 gives statistics for another LCA model which was alternatively developed to address the residual score for the local dependency between the Pap test and colposcopy/biopsy. Rather than adding another direct effect for this second local dependency, a 3-class model was generated maintaining the single direct effect for the dependency between VIA and colposcopy/biopsy. Figure 2 presents the conditional probabilities of the various test results given each class for this model. Note that this 3-class model with just one adjustment accounts for slightly more variance than did the 2-class model with two adjustments, having a higher likelihood (LL). Although the fit of this model to the data using LL appears slightly better than the 2-class model with two adjustments, the difference in the AIC statistics (12178.47 versus 12181.15) is very small (2.68), suggesting that the two models are similarly distant from a theoretical best model. That is, both models could be considered acceptable by the AIC criterion. Their relative usefulness, therefore, also needed to be evaluated in terms of biological plausibility (see Discussion).

As described earlier, sensitivity and specificity under the two models were calculated by recoding each test into its standard dichotomous categories. This was done by combining the appropriate conditional probabilities from Figures 1 and 2. For example, in the 3-class model, the sensitivity of VIA was 0.74 (probability of VIA = abnormal or cancer, given class 3). Specificity was 0.57. In that model, class 1 (p = 0.682) and class 2 (p = 0.200) combine to form non-disease. Specificity was calculated as 0.323 (probability of VIA inflammation given class 2) plus 0.117 (probability of VIA normal given class 2) multiplied by the probability of class 2 (0.200), plus the analogous values for Class 1, i.e., 0.429 (probability of VIA inflammation given class 1) plus 0.276 (probability of VIA normal given class 1) multiplied by the probability of class 1 (0.682).

Table 4 provides a summary of the prevalence and sensitivity and specificity values for all tests, for comparative purposes, for both the 2-class and 3-class LCA models. The first two rows are the conventionally-derived estimates of sensitivity and specificity for the three tests (for comparative purposes, with disease defined at two thresholds: HGSIL+ and LGSIL+). The last two rows represent the estimates for all tests from the two LCA models. The definition of positive for each test in the model is provided in the third column.

**Table 4:** Comparative values: conventional versus LCA model results

Reference standard	Disease prevalence (± SE)	Test positive	Sensitivity (± SE) (*)	Specificity (± SE)
1. Colposcopy/Biopsy LGSIL+	0.233 (0.009)	VIA Abnormal, CA	0.640 (0.022)	0.671 (0.012)
		Pap LGSIL +	0.298 (0.021)	0.923 (0.007)
		HPV >= 1.0 RLU	0.649 (0.022)	0.638 (0.012)
2. Colposcopy/Biopsy HGSIL+	0.096 (0.006)	VIA Abnormal, CA	0.775 (0.030)	0.639 (0.011)
		Pap LGSIL +	0.445 (0.035)	0.905 (0.007)
		HPV >= 1.0 RLU	0.800 (0.028)	0.613 (0.011)
3. LCA disease derived from Trichotomous ^(†) ^VIA, Pap, HPV, including colposcopy/biopsy: 2 class solution	0.302 (0.028)	VIA Abnormal, CA	0.637	0.701
		Pap LGSIL +	0.417	0.997
		HPV >= 1.0 RLU	0.973	0.807
		Colposcopy/Biopsy LGSIL+ ^(‡)^	0.442	0.857
		Colposcopy/Biopsy HGSIL+	0.251	0.970
4. LCA disease derived from Trichotomous ^(†) ^VIA, Pap, HPV, including colposcopy/biopsy: 3 class solution	0.118 ^(§)^ (0.022)	VIA Abnormal, CA	0.744	0.568
		Pap LGSIL+	0.560	0.820
		HPV >= 1.0 RLU	0.972	0.568
		Colposcopy/Biopsy LGSIL+	0.926	0.758
		Colposcopy/Biopsy HGSIL+	0.632	0.860

^(*) ^Standard errors (SE) are not given for the LCA-derived sensitivity and specificity estimates because some of these estimates are themselves calculated from combinations of conditional probabilities, which have individual maximum-likelihood estimated (MLE) standard errors. The MLE standard errors for these component conditional probabilities are given in Figures 1 and 2, in parentheses.

^(†) ^Trichotomous response codes are used to define the latent classes, but results are re-interpreted dichotomously in order to calculate sensitivity and specificity estimates.

^(‡) ^Results are given for Colposcopy/Biopsy with a low threshold (LGSIL+ is test positive, as it would be for Pap smears), and also with a high threshold (HGSIL+) as the latter is the threshold at which the gold standard for VIA testing is usually set when using the traditional formula.

^(§) ^Prevalence of class 3 (disease) in a 3-class model; all other LCA prevalence estimates are from 2-class models.

## Discussion

There are various aspects of study design and implementation that can affect the validity of test accuracy studies [39]. Regarding the accuracy of VIA as a test for (pre)cancer of the cervix, the majority of published studies to date have been conducted in low-resource settings. Differences in the threshold point defining VIA test positive, the threshold defining disease (LGSIL, HGSIL or cancer), the intensity and timing of provider training, the background experience and qualifications of the providers, sample sizes and sexually-transmitted disease risk of the participating women, among other factors, all could potentially account for differences in observed VIA accuracy estimates [40,41].

Two problems in particular – the sometimes variable quality of gold/reference standard tests and verification bias – have the potential to substantially negatively affect test accuracy results [42-44]. This analysis focused on clarifying the potential problem of imperfections in the gold/reference standard. It also addressed another purported issue with VIA accuracy studies, that is, the potential lack of independence between VIA and coloposcopy when the latter is used as, or part of, the reference standard.

In our opinion, the latent class model with three classes represents a more realistic assessment of the *true *sensitivities/specificities of VIA, Pap smear, and HC2 testing than do results from the conventional model with colposcopy/biopsy as the reference standard. This model showed good fit to the data and likely yields a more accurate study reference standard. The 3-class model offers a more stringent definition of disease, backed by three of the four tests (not the Pap test) and a prevalence rate of disease (CIN2+/HGSIL+ or cancer) for class three more consistent with that calculated using colposcopy/biopsy as the reference standard (around 10 percent). Prevalence values for the three latent classes in this model were 0.682 (SE = 0.029), 0.200 (SE = 0.035) and 0.118 (SE = 0.022) for latent disease class one, two, and three, respectively (Table 4). Given the high probabilities with more severe VIA, HC2, *and *colposcopy/biopsy results, class three in the 3-class model can more likely be interpreted as true "disease" (CIN2+/HGSIL+). This is supported by the high sensitivity rates for all tests in this class, as well as similar probability profiles for VIA and colposcopy/plus the high HC2 RLU levels (suggestive of high viral loads).

With this subset of Zimbabwe data, the LCA-derived sensitivity and specificity for VIA were fairly consistent with conventionally-derived estimates as well as the range of published values [4-13]. For comparative purposes, the LCA-derived sensitivity of HC2 was close to 97 percent for both the 3- and 2-class models. This is considerably higher than the conventionally derived estimate (0.80) and is more consistent with the ranges cited in some industrialized country meta-analyses [45]. However, other reviews indicate a slightly wider variation in HC2 sensitivity and reports from developing countries show a sensitivity lower than that commonly reported for developed countries [32,46-49]. In the recent scientific literature, the sensitivity of cytology at cutoff LGSIL+ for an outcome of CIN2+ ranged from 23% to 99% and the specificity from 7 to 97% [15]. Our LCA-derived cytology sensitivity and specificity differed slightly from the conventionally-derived values and fell within these published value ranges.

In this analysis, HC2 sensitivity showed the greatest change (gain) among the three tests comparing the conventional results to those using the latent reference standard with class three defining disease. Specificity for HC2, on the other hand, was relatively low for the 3-class model. Only the Pap test in this model continued to perform "counter-intuitively", with higher probabilities of latent disease class three for less severe Pap test results. However, this finding is consistent with the results from the initial, conventionally-derived cytology analysis that indicated a low ability for Pap smears in this setting to identify true disease [5].

Given the conditions of the Zimbabwe study, where the colposcopist was blinded to the results of any other test result, our LCA findings may reflect the more subjective nature of colposcopy and the consequences of a colposcopist seeing what they think is an insignificant lesion, for which they elect not to take a biopsy. The latent class model, which takes into account the additional information provided by the HC2, VIA and Pap results, classifies as true disease some lesions assessed on coloposcopy as "insignificant", rendering those colposcopy/biopsy results "false negatives". These translate into a reduced LCA-derived sensitivity value for the colposcopy/biopsy combined test.

Although the subjective nature of colposcopy has been commented on by many authors, the data from this exercise demonstrate the degree to which such subjectivity can potentially affect sensitivity or specificity estimates when a test is being evaluated with colposcopy/biopsy as a combined reference standard [50-52]. Mitchell et al (1998) summarized the sensitivity and specificity of colposcopy (compared to biopsy as the reference standard) through a meta-analysis of 9 studies [16]. They found a range of sensitivities from 0.30 – 0.99 and a range of specificities from 0.39 – 0.93, with a weighted mean sensitivity and specificity of 0.85 and 0.69, respectively. All this suggests that colposcopy may have limitations when used as a "reference" standard, alone or in combination with biopsy, for cervical cancer test accuracy studies.

In this study, despite apparent imperfections in the reference standard, the conventionally-derived VIA results fell within the range of published data and were relatively consistent between with the 3-class LCA model (0.775 versus 0.744, respectively, for sensitivity and 0.639 and 0.568, respectively for specificity). HC2 in this study however proved to have higher sensitivity when measured using LCA. This may explain, in part, the discrepancy observed between study results from industrialized countries versus those originally from Zimbabwe [32]. However, as noted earlier, lower HC2 sensitivity has also been observed in other developing country studies [47-49]. This could similarly be due to inadequacies in the gold standard used or to imperfections in sample collection, transport or processing or a combination.

The age range of the women (22–55) was limited in this study for two important reasons. First, because large numbers of women can be infected with HPV but not have persistent disease, we wanted to maximize chances that any identified lesions would likely represent real disease versus squamous metaplasia, inflammation or transient infection with HPV. Second, as women age, especially when they become post-menopausal, the squamo-columnar junction (which is used as an anatomical landmark for VIA assessments) recedes into the cervical canal and sometimes cannot be visualized. In such women, VIA is likely to be incomplete or unsatisfactory affecting the accuracy of the test. This study criterion limits the generalizablity of the results to the population of women of the same age range. However, for developing countries seeking more affordable cancer prevention strategies, given the natural history of disease and the intrinsic limitations of VIA among older women, it has been shown that focusing on this age range is cost effective [53].

## Conclusion

To have greater confidence in results of conventional accuracy studies for existing or proposed cervical cancer screening tests, especially where there are questions about the reference standard, analyses involving LCA merit more attention. In this particular setting, LCA yielded accuracy estimates which, after adjustment, fell within the range of values observed in studies where high quality colposcopy and biopsy were used [45,46]. Additionally, using LCA it was possible to account for any correlation between VIA and colposcopy which both rely on visual interpretation of the cervix after acetic acid application. If not adjusted for, this dependency between the test and the gold standard artificially inflates sensitivity [31].

LCA however also has its limitations as a "diamond" standard is ultimately required to verify the LCA truth [54]. Under this approach, disease is not formally defined but rather latent disease (truth) is a mathematically defined entity that does not necessarily correspond with a clinically relevant status. Moreover, LCA modeling requires specification of the joint distribution of test results, conditional on disease status. The model however cannot be fully tested with the observed data. Statistical associations between tests therefore should be understood biologically otherwise the meaning of the resulting estimates may be unclear [22]. Consequently, researchers designing screening/diagnostic test studies should designate resources for verifying true disease outcome using an improved gold standard on at least a representative subset of study subjects [55]. Consideration can also be given to alternative approaches to evaluating diagnostic/screening test accuracy (e.g., a composite reference standard) when a gold standard does not exist [56,57].

Additionally, given that a perfect reference standard for cervical cancer may be unattainable, even in a controlled clinical setting, efforts to determine the relative usefulness of new tests should also consider how consistent study results are with the weight of existing data rather than trying to identify a single, best "truth". In this regard, a recent cost-effectiveness analysis of cervical cancer screening strategies showed that (under model assumptions, including industrialized country accuracy values for HC2) VIA, with immediate treatment for test-positive women at first visit, was similarly effective in reducing cancer incidence over the lifetime of the simulated cohort as HC2 screening with treatment at a second followup visit (26 percent versus 27 percent cancer incidence reduction, respectively). The HC2-based approach was less *cost*-effective, however, than VIA with the immediate option of treatment – the main factor being the number of women in resource-limited environments who often drop out when more than one visit linking testing and treatment is involved. Cytology, followed by treatment of test-positive women at a second visit, was the least effective (19 percent cancer incidence reduction) and the least cost-effective [53].

In 1994, the World Health Organization recommended exploring the benefits of VIA as an alternative screening test for cervical cancer in underserved developing countries [58]. This study confirms the accuracy of VIA in detecting lesions requiring treatment at the hands of nurse-midwives in such a low resource setting. This finding, plus the fact that VIA is simple to administer, can be performed by nurse-midwives and the results are immediately available, make it a particularly valuable option for many resource-poor settings.

## Competing interests

The author(s) declare that they have no competing interests.

## Authors' contributions

LG conceived and coordinated the study, participated in its design, participated in interpretation of the results and oversaw the development of the manuscript. JAM participated in the study design, performed the statistical analyses, participated in the interpretation of the results and helped to finalize the manuscript. MA aided in determining statistical analyses, participated in the interpretation of the results and helped to finalize the manuscript. PDB participated in the study design, participated in interpretation of the results and helped to finalize the manuscript. All authors read and approved the final manuscript.

## Pre-publication history

The pre-publication history for this paper can be accessed here:


